# A Case Report on Scrofuloderma: A Cutaneous Manifestation of Tuberculosis

**DOI:** 10.7759/cureus.62565

**Published:** 2024-06-17

**Authors:** Soham R Meghe, Adarshlata Singh, Drishti M Bhatt, Shreya N Gupta, Varun Hanumanthaiah, Shree Ramya Talasila

**Affiliations:** 1 Dermatology, Jawaharlal Nehru Medical College, Datta Meghe Institute of Higher Education and Research, Wardha, IND

**Keywords:** chronic granulomatous inflammation, skin tuberculosis, cutaneous manifestation, tuberculosis, scrofuloderma

## Abstract

Scrofuloderma, a manifestation of cutaneous tuberculosis, is a less common but clinically significant form of mycobacterial infection. It typically arises from the contiguous spread of *Mycobacterium* *tuberculosis* from an underlying infected lymph node or osseous structure to the adjacent skin. The condition manifests predominantly as chronic, granulomatous inflammation that leads to skin ulcers and abscesses. Despite its rarity, scrofuloderma presents substantial diagnostic challenges, primarily due to its nonspecific clinical presentation, which may mimic other dermatological conditions. This case report delineates the clinical journey of a patient with scrofuloderma who was attended to in a tertiary care setting. It emphasizes the diagnostic complexities encountered, underscored by a comprehensive discussion of the investigative modalities used to establish the diagnosis. This report elaborates on the therapeutic regimen taken, showcasing the effectiveness of a customized antituberculosis treatment plan.

## Introduction

Tuberculosis (TB), caused by *Mycobacterium* *tuberculosis*, remains one of the most significant infectious diseases worldwide, posing a substantial public health challenge. Although primarily known for its pulmonary manifestations, TB can affect virtually any organ system in the body, leading to a wide range of clinical presentations [1]. Among these, cutaneous TB represents a less common but clinically important form, encompassing various dermatological manifestations resulting from mycobacterial infection of the skin. Scrofuloderma, also known as tuberculosis cutis colliquativa, is a distinct form of cutaneous TB that typically arises from the direct extension of an underlying TB infection of the lymph nodes, bones, or other organs to the skin [2]. Scrofuloderma TB, the central focus of this article, represents a rare but clinically significant manifestation of cutaneous TB. While TB remains a major global health concern, with an estimated 10.6 million new cases and 1.6 million deaths reported globally in 2021, scrofuloderma constitutes a small fraction of these cases, highlighting its rarity [3].

Despite significant advancements in TB diagnostics and treatment, particularly in resource-limited settings where access to healthcare is often limited, scrofuloderma presents diagnostic challenges due to its infrequency and nonspecific clinical presentation [4]. Moreover, the emergence of multidrug-resistant strains of *Mycobacterium tuberculosis* further complicates the treatment landscape, underscoring the importance of identifying and treating all forms of the disease, including its cutaneous manifestations [5]. While specific prevalence data for scrofuloderma TB may be scarce due to its rarity, case reports and relevant literature document sporadic occurrences, emphasizing the need for heightened clinical suspicion and awareness of this condition.

Scrofuloderma is most commonly observed in children and young adults because the early life stages are when most immunologically immature infectious agents occur, and it often indicates an underlying TB infection that may not have been previously diagnosed. The development of scrofuloderma reflects the response to the mycobacterial infection, resulting in chronic granulomatous lesions that eventually ulcerate and discharge through the skin. This process not only leads to significant morbidity for the affected individual but also poses a risk of transmission to others, highlighting the importance of early diagnosis and treatment [2]. The pathogenesis of scrofuloderma involves the breakdown of the skin barrier due to the direct extension of an underlying TB focus, such as an infected lymph node or bone. This extension is facilitated by the body's immune response to infection, which attempts to contain the spread of mycobacteria but, in doing so, leads to inflammation and necrosis of the affected tissues [6]. The clinical presentation of scrofuloderma is characterized by the appearance of nodules that gradually enlarge, become fluctuant, and eventually rupture, forming ulcers that discharge a characteristic seropurulent material. These lesions are often accompanied by systemic symptoms of TB, such as fever, weight loss, and malaise. In some cases, the cutaneous manifestations can precede the recognition of systemic involvement [7,8].

The diagnosis of scrofuloderma is based on a combination of clinical findings, histopathological examinations, microbiological studies, and, when applicable, imaging studies to assess the extent of systemic involvement. Histopathologically, scrofuloderma is characterized by granulomatous inflammation with or without caseation necrosis, a hallmark of TB infection [7]. In addition to the diagnostic challenges posed by scrofuloderma, it is imperative to consider other granulomatous diseases in the differential diagnosis due to overlapping clinical and histopathological features. Granulomatous diseases such as sarcoidosis, leishmaniasis, fungal infections (e.g., histoplasmosis, sporotrichosis), and other mycobacterial infections (e.g., atypical mycobacteria) can present with similar cutaneous manifestations, complicating the differentiation from scrofuloderma.

Sarcoidosis, characterized by non-caseating granulomas, may mimic cutaneous TB, with systemic symptoms aiding differentiation. Leishmaniasis, caused by protozoan parasites, can resemble scrofuloderma; travel history and serological tests are helpful in diagnosis. Fungal infections such as histoplasmosis, sporotrichosis, and atypical mycobacterial infections pose similar challenges; histopathology, cultures, and molecular techniques aid in differentiation.

In endemic areas, a high index of suspicion and multidisciplinary collaboration are crucial for accurate diagnosis and optimal management. [9]. The management of scrofuloderma involves a multidisciplinary approach, including dermatologists, infectious disease specialists, and sometimes surgeons, depending on the extent of the disease. The cornerstone of treatment is antituberculosis therapy, following the standard protocols for treating TB. The duration and composition of the treatment regimen may vary depending on the susceptibility of the *Mycobacterium tuberculosis* strain drug, the patient's clinical response, and the presence of drug-resistant TB [7]. Treatment not only aims to eradicate the mycobacterial infection but also to prevent the spread of the disease to others and to minimize long-term sequelae, such as scarring and disfigurement, that can result from cutaneous manifestations of TB.

## Case presentation

A 35-year-old male patient with no significant medical history presented to the dermatology outpatient department with a progressive, painless swelling of his neck that had been developing over five months. The patient was treated in a tertiary care center in central India. Initially noticed as a small, firm nodule, the lesion gradually increased, eventually ulcerating and discharging a serous fluid. Despite the progression of the lesion, the patient reported no systemic symptoms such as fever, night sweats, weight loss, or persistent cough.

**Figure 1:**
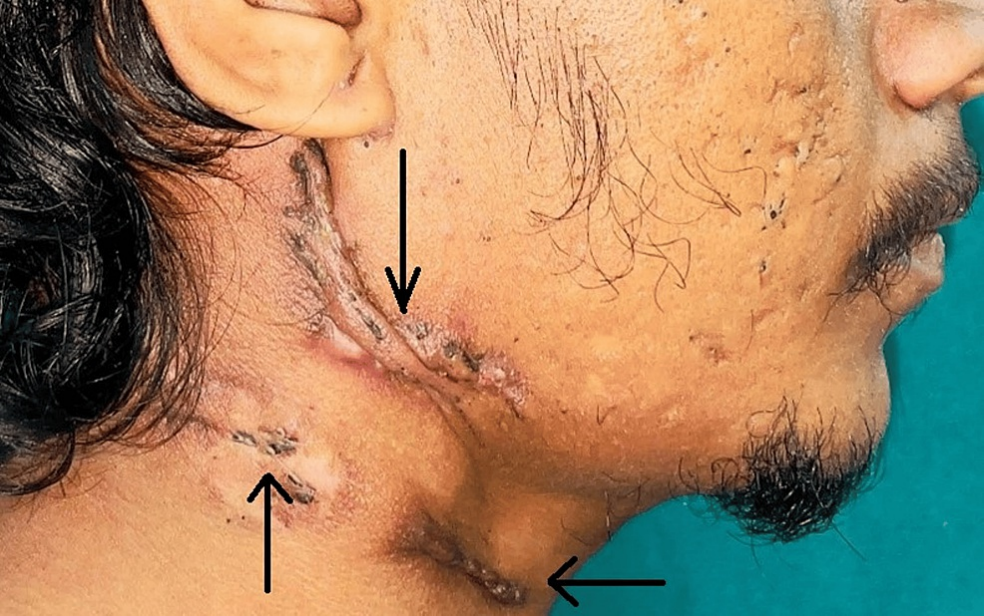
Lesions with pus-discharging sinuses on the neck The black arrows show black-colored ulcerative lesions present with pus-discharging sinuses with puckered scar marks on the neck

Figure 1 shows a large confluent nodule on the neck, with parts of the overlying skin appearing violaceous and others showing signs of ulceration. Some areas of the lesion had broken down to form sinuses that discharged a seropurulent material. No other similar lesions were observed elsewhere in the body. The patient denied any known contact with people diagnosed with active TB.

Investigations

In the investigation phase, the patient underwent a comprehensive series of tests to confirm the diagnosis of scrofuloderma. An incisional skin biopsy revealed granulomatous inflammation with caseating necrosis, indicative of TB. Despite the absence of pulmonary involvement as demonstrated by a normal chest X-ray and computed tomography (CT) scan, the patient's Mantoux test was positive with an induration of 15 mm, suggesting a latent or active mycobacterial infection. Table 1 shows the cartridge-based nucleic acid amplification test (CBNAAT) on the material discharged from the lesions that identified the low presence of *Mycobacterium tuberculosis*, further confirming the cutaneous manifestation of TB.

**Table 1:** CBNAAT test report CBNAAT: cartridge-based nucleic acid amplification test; QC: quality control; Ct: cycle threshold; NA: not applicable; SPC: sample processing control; POS: positive; NEG: negative

Analyte Name	Ct	EndPt	Analyte Result	Probe Check Result
Probe D	23.8	173	POS	PASS
Probe C	23.5	176	POS	PASS
Probe E	24.8	113	POS	PASS
Probe B	24.0	106	POS	PASS
SPC	22.9	265	NA	PASS
Probe A	23.2	110	POS	PASS
QC-1	0.0	0	NEG	PASS
QC-2	0.0	0	NEG	PASS

Table 2 shows the patient's results and indicates the findings in various parameters. In the complete blood count (CBC), the hemoglobin level (Hb%) was 10.8 g/dL. The mean corpuscular hemoglobin concentration (MCHC) was 32.2 g/dL, while the mean corpuscular volume (MCV) was lower at 71.3 fL. The red blood cell (RBC) count was 4.72 x 10^12^/L, and the white blood cell (WBC) count was elevated at 10100 cells/μL. The platelet count was slightly low at 4.32 x 10^9^/L, below the normal range. In the coagulation profile, the activated partial thromboplastin time (APTT) was prolonged to 52.3 seconds, while the prothrombin time (PT) and the international normalized ratio (INR) were within normal limits. The peripheral smear indicates a normocytic normochromic picture. The thyroid function tests reveal normal levels of free triiodothyronine (FT3) and free thyroxine (FT4), along with thyroid-stimulating hormone (TSH), within the normal range. Kidney function tests showed urea and creatinine levels within normal limits. Liver function tests demonstrate levels of alanine aminotransferase (ALT) and aspartate aminotransferase (AST) within the normal range and total bilirubin levels. Random blood sugar was slightly elevated at 123 mg/dL. Virology tests for hepatitis B surface antigen (HBsAg), hepatitis C virus (HCV), and human immunodeficiency virus (HIV) were negative. Collectively, these investigations supported the diagnosis of scrofuloderma, facilitating the initiation of targeted antituberculosis therapy.

**Table 2:** Clinical report of the patient Hb: hemoglobin; MCHC: mean corpuscular hemoglobin concentration; MCV: mean corpuscular volume; RBC: red blood cell; WBC: white blood cell; APTT: activated partial thromboplastin time; PT: prothrombin time; INR: international normalized ratio; FT3: free triiodothyronine; FT4: free thyroxine; TSH: thyroid-stimulating hormone; ALT: alanine aminotransferase; AST: aspartate aminotransferase; SGOT: serum glutamic-oxaloacetic transaminase; SGPT: serum glutamate pyruvate transaminase; HBsAg: hepatitis B surface antigen; HCV: hepatitis C virus; HIV: human immunodeficiency virus; pg/mL: picograms per milliliter; ng/mL: nanograms per deciliter; μIU/mL: micro-international units per milliliter; mg/dL: milligrams per deciliter; U/L: units per liter; g/dL: grams per deciliter; fL: femtoliters

Test	Patient's Value	Reference Value
Hb	10.8 g/dL	12-15 g/dL
MCHC	32.2 g/dL	32-36 g/dL
MCV	71.3 fL	80-100 fL
RBC count	4.72	4.5-5.5x10^12^/L
WBC count	10100 cells/μL	4000-11000 cells/μL
Platelet count	4.32x10^9^/L	150-450x10^9^/L
APTT	52.3 seconds	30-40 seconds
PT	13.5 seconds	11-14 seconds
INR	1.14	0.8-1.2
Peripheral smear	Normocytic normochromic	N/A
FT3	3.94 pg/mL	2.0-4.4 pg/mL
FT4	1.59 ng/dL	0.93-1.7 ng/dL
TSH	2.33 μIU/mL	0.27-4.2 μIU/mL
Urea	20 mg/dL	10-50 mg/dL
Creatinine	0.9 mg/dL	0.6-1.2 mg/dL
ALT (SGPT)	18 U/L	< 35 U/L
AST (SGOT)	22 U/L	< 35 U/L
Total bilirubin	0.5 mg/dL	0.1-1.2 mg/dL
Random blood sugar	123 mg/dL	< 140 mg/dL
HBsAg	Negative	N/A
HCV	Negative	N/A
HIV	Negative	N/A

**Figure 2:**
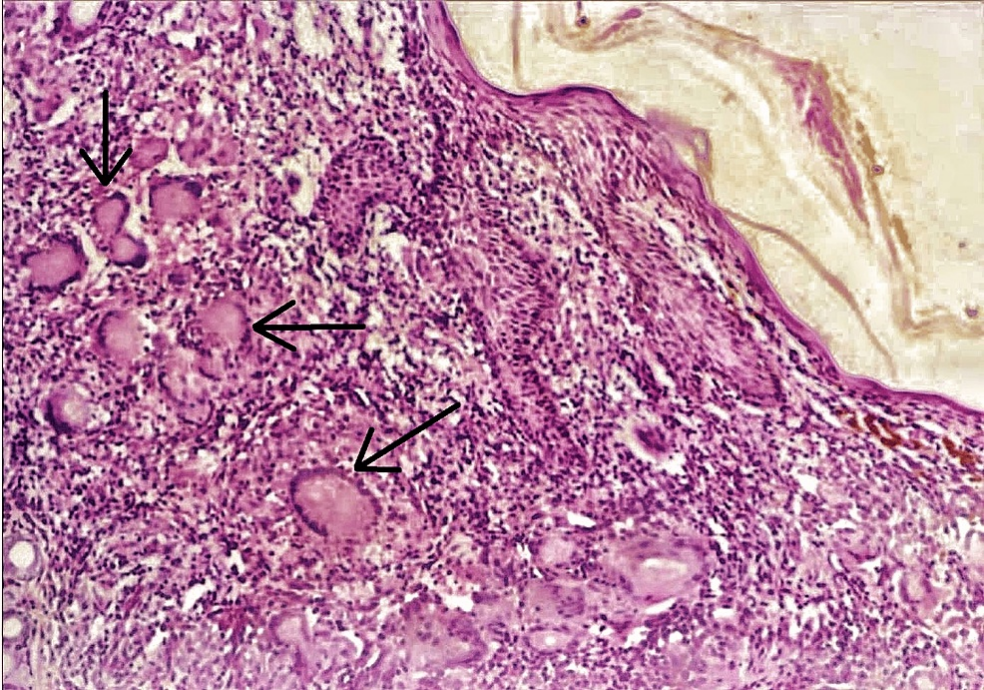
Histopathological image of the scrofuloderma The black arrows show Langhans-type giant cells with a typical "horseshoe" arrangement of the nuclei

Figure 2 shows the section of scrofuloderma stained with routine hematoxylin and eosin, showing decreased thickness of the epidermis with alterations in the underlying tissue, showing caseating necrosis, Langhans-type giant cells with a typical "horseshoe" arrangement of the nuclei, and epithelioid histiocytes surrounded by mononuclear cells.

Treatment

Antitubercular treatment was immediately started. The regimen consisted of a fixed dose combination of first-line antitubercular drugs that are tab isoniazid 75 mg, tab rifampicin 150 mg, tab pyrazinamide 400 mg, and tab ethambutol 275 mg administered four tabs once a day (based on the weight band of the patient) for the first eight weeks (Intensive phase). This was followed by a continuation phase drug consisting of a 75 mg tab of isoniazid, a 150 mg tab of rifampicin, and a 275 mg tab of ethambutol, lasting 16 weeks to eradicate and prevent the development of multidrug-resistant (MDR) TB. Meticulous wound care was provided for ulcerated skin lesions, focusing on promoting healing and preventing secondary infections. This treatment strategy underscores the importance of a multidisciplinary approach, integrating pharmacological therapy with supportive care, to achieve optimal results in managing scrofuloderma.

Outcome and follow-up

The patient demonstrated a notable clinical improvement within the initial two months of antituberculosis therapy, evidencing a significant reduction in lesion discharge and the beginning of healing in the ulcerated areas.

## Discussion

Skin infection next to a tuberculous focus causes scrofuloderma, which can be related to peripheral ganglion TB or TB of the bone, joint, or testicles. Subcutaneous, painless, slowly growing nodules that develop into ulcers and fistulous tracts with serous, purulent, or caseous substances draining from them are the hallmarks of the clinical presentation [7]. This case highlights how crucial it is to rule out TB as a differential diagnosis when patients appear with persistent, chronic skin lesions, especially in areas where the disease is still highly prevalent.

Lupus vulgaris, tuberculosis verrucosa cutis, tuberculous chancre, and tubercular gumma are distinct forms of cutaneous TB caused by direct skin infection by *Mycobacterium tuberculosis*. These conditions are not considered tuberculides, hypersensitivity reactions to TB elsewhere in the body. Tuberculides include erythema induratum (nodular vasculitis), lichen scrofulosorum, and papulonecrotic tuberculid. Scrofuloderma, on the other hand, is unique in that our patient presented with asymptomatic swellings that persisted for months before becoming soft and ulcerating. This manifestation arises from infection and skin deterioration above a superficial tuberculous focus, typically affecting lymph nodes, particularly those in the cervical chain [10]. Despite the absence of systemic symptoms such as fever, weight loss, or chronic cough, which are often associated with active TB, the patient showed a positive Mantoux test and verified mycobacterial presence in the lesion discharge, supporting the diagnosis of cutaneous TB.

Umapathy et al. [11] stated in their study that the drug resistance pattern of the isolated tubercle bacilli in their study, including initial multi-drug resistance (to isoniazid and rifampicin), was comparable to the resistance pattern observed in newly admitted patients with pulmonary TB to various controlled clinical trials at their center [11]. A combination of antitubercular medications is used to treat scrofuloderma to successfully remove the mycobacterial infection and avoid drug resistance or recurrence of the condition. Four-drug therapy proved to be a viable treatment strategy for our patient despite the absence of MDR TB in our case.

## Conclusions

In conclusion, this case underscores the imperative to consider TB in the spectrum of granulomatous skin diseases, advocating for prompt diagnosis, efficacious treatment strategies, and the mitigation of potential complications. Moreover, it highlights the pivotal role of early detection, precise diagnosis, and meticulous management in ameliorating patient outcomes and mitigating the burden of TB-related skin manifestations. Notably, the uniqueness of this case lies in its contribution to the existing body of knowledge by elucidating the diagnostic and therapeutic challenges encountered in managing scrofuloderma. By delineating the clinical course and outcomes, this report offers novel insights into the complexities of TB-associated cutaneous manifestations. Thus, it accentuates the significance of continued vigilance, interdisciplinary collaboration, and innovative approaches in confronting TB-related dermatological conditions.
